# A network analysis of the relationship between temperament characteristics and problem behaviors in preadolescents

**DOI:** 10.3389/fpsyg.2026.1854621

**Published:** 2026-06-24

**Authors:** Fang Liu, Jiani Gao, Hanqi Li, Hanbo Che, Linlin Lin

**Affiliations:** 1 Department and Institute of Psychology, Ningbo University, Ningbo, Zhejiang, China; 2 School of Education, Liaoning Normal University, Dalian, Liaoning, China; 3 Ningbo Childhood Education College, Ningbo, Zhejiang, China

**Keywords:** aggressive behavior, depression, network analysis, problem behaviors, temperamental characteristics

## Abstract

Temperament, defined as relatively stable, innate tendencies in emotional reactivity and self-regulation, is a robust correlate of childhood problem behaviors. However, while most studies focus on isolated symptoms, few have examined how specific temperamental traits simultaneously relate to the complex interactions between internalizing and externalizing systems. Using network analysis, this study explored the relationships among temperamental traits, internalizing problems (depressive symptoms), and externalizing problems (aggressive behavior) based on self-report data from 642 preadolescents. We estimated a Gaussian Graphical Model via the EBICglasso approach, treating 12 temperament, 5 depression, and 4 aggression dimensions as nodes, and subsequently calculated their Expected Influence (EI) and Bridge Expected Influence (BEI). Results revealed that negative self-esteem emerged as the most central node, followed by displaced aggression and negative mood (Depressive Symptoms). In terms of bridge centrality, depressive mood (Temperament) exhibited the highest bridge expected influence, emerging as the primary bridge node connecting the internalizing and externalizing systems, followed by hostility and displaced aggression, which served as significant bridge hubs connecting the temperament, depression, and aggression communities. Collectively, our findings provide an initial characterization of the conditional association patterns among these variables.

## 1 Introduction

Preadolescence constitutes a defining developmental nexus between childhood and adolescence. During this critical window, internalizing problems (e.g., depression) and externalizing problems (e.g., aggression) exert a dual burden, which acutely disrupt preadolescents’ academic progress and social integration while concurrently serving as robust antecedents for severe mental disorders in later life. Crucially, this transitional phase is marked by heightened sensitivity to environmental influences, during which individual differences in temperament actively interact with these problems, such that temperamental traits are associated with the expression of these problems, with their severity, and may be linked to the trajectory of psychopathology. Thus, clarifying how temperament and preadolescent problem behaviors are connected is an essential step toward understanding which patterns of association may be most relevant for future research on risk and prevention (Franzoi et al., 2024; Lee et al., 2024). Temperament—defined as innate, relatively stable tendencies in emotional and behavioral reactivity—has been established as a fundamental early antecedent explaining the emergence and comorbidity of problem behaviors (Shiner, 2012). However, most existing studies have examined the associations between temperament and problem behaviors in isolation, leaving a lack of systematic investigation into how temperamental traits simultaneously relate to the complex interplay between internalizing and externalizing systems. Therefore, focusing on preadolescence as a sensitive developmental juncture, the present study employs network analysis to systematically elucidate the complex network structure linking preadolescents’ temperamental characteristics to depression and aggressive behavior, as well as the patterns of association within it.

Temperament, as defined by Rothbart’s model (2011), comprises three core dimensions—negative emotionality (tendency to distress), extraversion/surgency (approach motivation), and effortful control (self-regulation)—that are theorized to be closely linked to the development of internalizing and externalizing problems. High negative emotionality predisposes to emotional overreactivity, which has been found to predict both withdrawal/depression and externalizing problems (Harris et al., 2025; Wang et al., 2021). Low effortful control undermines impulse inhibition, predisposing to impulsive and disruptive behaviors (Olson et al., 2017). Conversely, unchecked extraversion/surgency fosters risk-taking and aggression (Eisenberg et al., 2009). Together, these dimensions may jointly contribute to problem behavior trajectories through their differential associations with emotional reactivity, cognitive appraisals, and behavioral regulation in preadolescence.

The present study focuses on preadolescents, marking a critical transition from childhood to adolescence. During this phase, preadolescents’ social cognitive abilities develop rapidly, with marked improvements in perspective-taking and empathy. However, their emotion regulation capacities, especially effortful control-related attentional shifting and inhibitory control, remain immature (Eisenberg, 2005; Willner et al., 2022). Concurrently, peer relationships become increasingly salient, sensitivity to social evaluation rises sharply, and academic pressure intensifies (Cheng et al., 2024; Grunewald et al., 2022). This developmental mismatch between high reactivity and low regulation makes preadolescents prone not only to inward-directed depressive symptoms (e.g., low self-esteem, anhedonia) but also to outward-directed hostile attributions and aggressive behavior when facing interpersonal conflicts or academic setbacks (Chen and Wang, 2025). Therefore, during this dynamic transitional period, the connections between temperamental traits and internalizing/externalizing problems may exhibit a unique network pattern. Identifying the core nodes and bridge nodes within this network is particularly crucial for early intervention.

However, previous studies have typically focused on single outcomes, examining how temperament predicts either internalizing or externalizing problems in isolation (All and Huang-Pollock, 2025). Few have systematically explored how temperamental traits simultaneously influence both problem systems and shape their covariation. Furthermore, traditional linear models (e.g., regression and structural equation modeling) cannot adequately map the complex interactive networks between specific temperamental dimensions and problem behaviors. To overcome this limitation, the network theory of psychopathology offers a framework focused on direct variable associations (Borsboom and Cramer, 2013). Network analysis treats observed variables (e.g., specific temperament, depression, and aggression dimensions) as nodes. Using the EBICglasso approach, it estimates unique connections (edges) to directly visualize conditional dependencies among these variables (Briganti et al., 2024). Within this framework, Expected Influence (EI) quantifies a specific node’s overall centrality in the network. Meanwhile, Bridge Expected Influence (BEI) identifies crucial bridge nodes that connect distinct communities across different dimensions (Jones et al., 2021; Robinaugh et al., 2016).

Accordingly, the present study applied network analysis to systematically explore how multidimensional childhood temperament relates to internalizing (measured by depressive symptoms) and externalizing (measured by aggressive behavior) problems within a complex network structure. Specifically, we aimed to: (1) identify variables with the highest relative centrality to pinpoint the most central nodes in the overall temperament–problem behavior network; (2) locate bridge nodes connecting distinct variable communities (i.e., temperament, internalizing, and externalizing problems), thereby revealing the patterns of association that characterize covariation among co-occurring problem behaviors. We expect this study to offer novel insights into the complex patterns of association between temperament and problem behaviors.

## 2 Methods

### 2.1 Participants

In this study, preadolescents from grades 3 to 6 at a public elementary school in Dalian, Liaoning Province, China, were recruited using a convenience sampling approach. A total of 652 questionnaires were collected. Through listwise deletion, ten preadolescents with missing data were excluded. The final valid sample comprised 642 participants (52.3% boys, *n* = 336; 47.7% girls, *n* = 306) aged 8 to 12 years (*M* = 9.82, *SD* = 1.20), with no remaining missing values. This sample size (*N* = 642) is adequate for estimating a network model with 21 core observed nodes, as it effectively controls false positives and ensures sufficient statistical power.

### 2.2 Measures

#### 2.2.1 Temperament

The short self-report form of the Early Adolescent Temperament Questionnaire–Revised (EATQ–R) was developed by Ellis and Rothbart (2001). The scale comprises 12 dimensions: activation control, affiliation, attention, inhibitory control, fear, frustration, high-intensity pleasure, perceptual sensitivity, pleasure sensitivity, shyness, aggression, and depressive mood (Temperament). Items are rated on a 5-point Likert scale (1 = “Completely untrue” to 5 = “Completely true”), with some items reverse-scored (e.g., “It is very hard for me to finish tasks on time”). Higher scores reflect greater endorsement of the corresponding temperament trait. The EATQ–R has demonstrated satisfactory validity and reliability in cross-cultural contexts. In this study, the questionnaire showed good internal consistency, with a Cronbach’s *α* of 0.81 for the total score.

#### 2.2.2 Internalizing problems: depressive symptoms

The Chinese revised version of the Children’s Depression Inventory (CDI-C), adapted from the original scale developed by Kovacs (1985) and revised by Wu et al. (2010), was used to assess the core indicators of internalizing problems. The scale consists of five subscales: anhedonia, negative mood, negative self-esteem, ineffectiveness, and interpersonal problems. Items are rated on a three-point scale from 0 to 2 (0 = absent, 1 = mild, 2 = severe). Although the original scale contains 27 items, one item assessing suicidal ideation was removed in compliance with ethical guidelines, resulting in a final 26-item measure. Higher total and subscale scores indicate greater severity of depression. In this study, the Cronbach’s *α* was 0.84.

#### 2.2.3 Externalizing problems: aggression

The Chinese revision of the Buss-Perry Aggression Questionnaire (BPAQ) was adapted from the version developed by Liu et al. (2009) to assess indicators of externalizing problems. The scale consists of four dimensions: physical aggression, displaced aggression, anger, and hostility. The questionnaire contains 20 items tailored to the linguistic and cultural norms of Chinese preadolescents and adolescents, rated on a 5-point Likert scale (1 = “Strongly disagree” to 5 = “Strongly agree”). Higher scores indicate more severe aggression and externalizing problems. The revised scale has demonstrated good reliability and satisfactory validity among Chinese school-aged preadolescents. In this study, the questionnaire demonstrated good internal consistency, with a Cronbach’s *α* of 0.90.

### 2.3 Procedure

Data collection was conducted on a class-by-class basis using paper-and-pencil questionnaires. Before completing the measures, trained research assistants explained the research purposes and procedures in detail, and informed consent was obtained from both the preadolescents and their legal guardians. To minimize common method bias associated with self-reports, clear and standardized instructions were provided. The absolute anonymity and strict confidentiality of the study were emphasized before data collection. Participants were explicitly told that there were no right or wrong answers and were encouraged to respond based on their true feelings. They independently completed the questionnaires in a quiet classroom, which took approximately 40 min. Trained research assistants were present throughout to offer assistance, monitor for any confusion, and answer questions as needed. Upon completion, a brief explanation of the study was given to the preadolescents and their guardians, and small gifts were distributed as a token of gratitude.

### 2.4 Data analysis

Data preprocessing and descriptive statistics were performed using IBM SPSS Statistics 27.0, while network analysis was conducted in the R environment. To control for potential demographic variables, gender, age, and grade were included as covariates in the analysis. To account for the ordinal Likert-scale data, a polychoric correlation matrix was computed as the input for the EBICglasso network estimation (Flora and Curran, 2004; Johal and Rhemtulla, 2023).

The network model was estimated using the graphical lasso based on the Extended Bayesian Information Criterion (EBICglasso), utilizing the bootnet and qgraph packages. The model comprised 21 observed nodes, including 12 temperament dimensions, 5 depression dimensions, and 4 aggression dimensions. The EBICglasso algorithm effectively controls for false positives by shrinking spurious partial correlations to zero. Furthermore, a case-dropping bootstrap procedure (2,500 bootstraps) was used to test the robustness of the network’s centrality indices, and the correlation stability coefficient (CS-coefficient) was calculated. According to established guidelines, the CS-coefficient should be greater than 0.25, and ideally exceed 0.50, to ensure the robustness of the network structure.

Additionally, two centrality indices were computed using the bootnet package: (1) EI, defined as the sum of all edge weights connecting a specific node to all other nodes in the network, quantifies the node’s overall connectivity; and (2) BEI, which measures the connection strength between a node and nodes belonging to other communities (i.e., the temperament, depression, and aggression systems). BEI was used to identify crucial hubs that bridge different communities (i.e., temperament, depression, and aggression systems).

## 3 Results

All network models reported below were estimated from a polychoric correlation matrix via the EBICglasso algorithm with the tuning parameter set to *γ* = 0.5.

### 3.1 Robustness test

**Figure 1:**
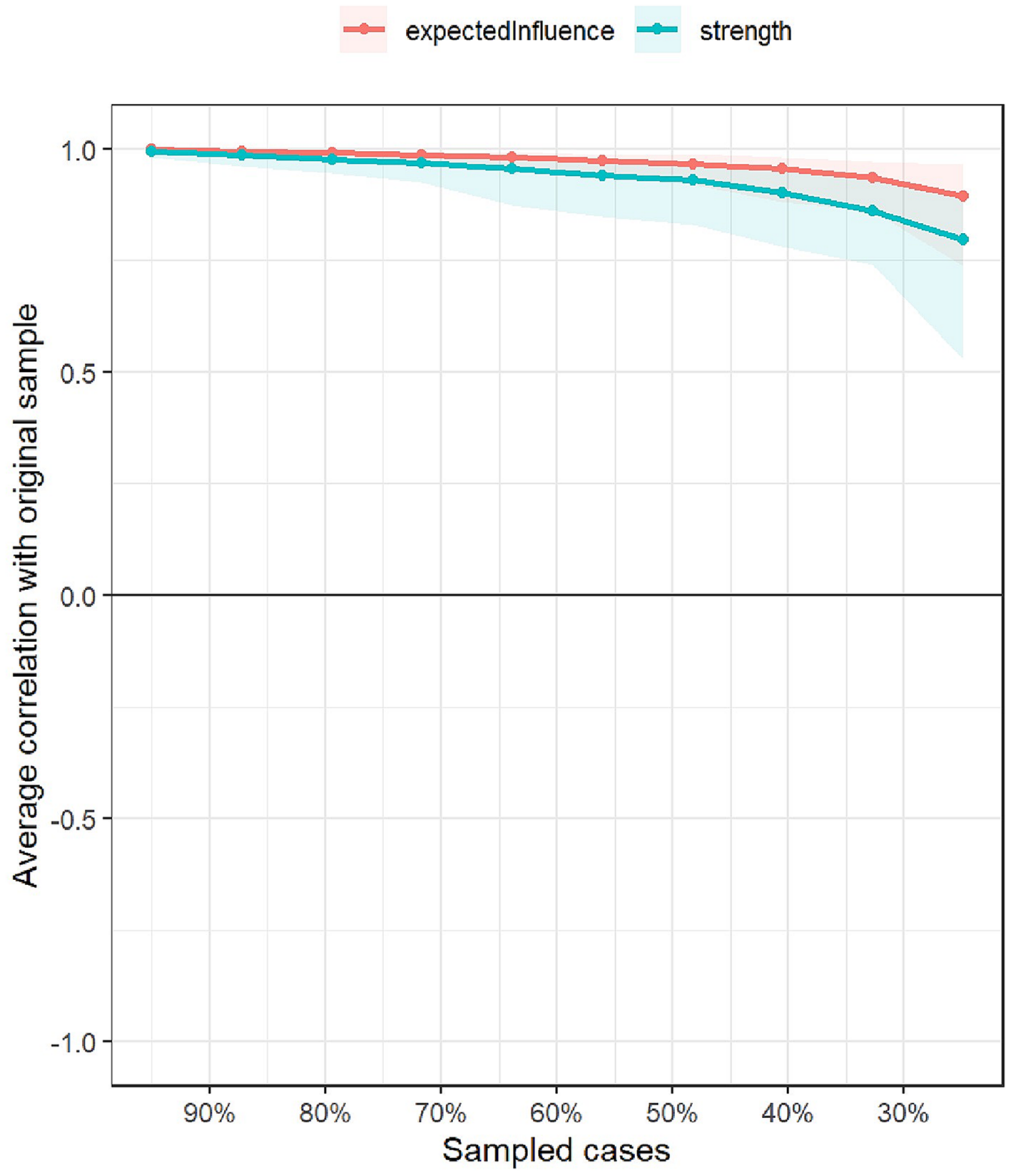
Stability test of centrality indices.

A case-dropping bootstrap procedure was employed to test the robustness of the node centrality indices. As shown in Figure 1, the CS-coefficients was 0.75, well above the recommended threshold of 0.50. These results demonstrate the high stability of the estimated network structure, indicating that the centrality indices are robust to outliers.

### 3.2 Network structure analysis

**Figure 2:**
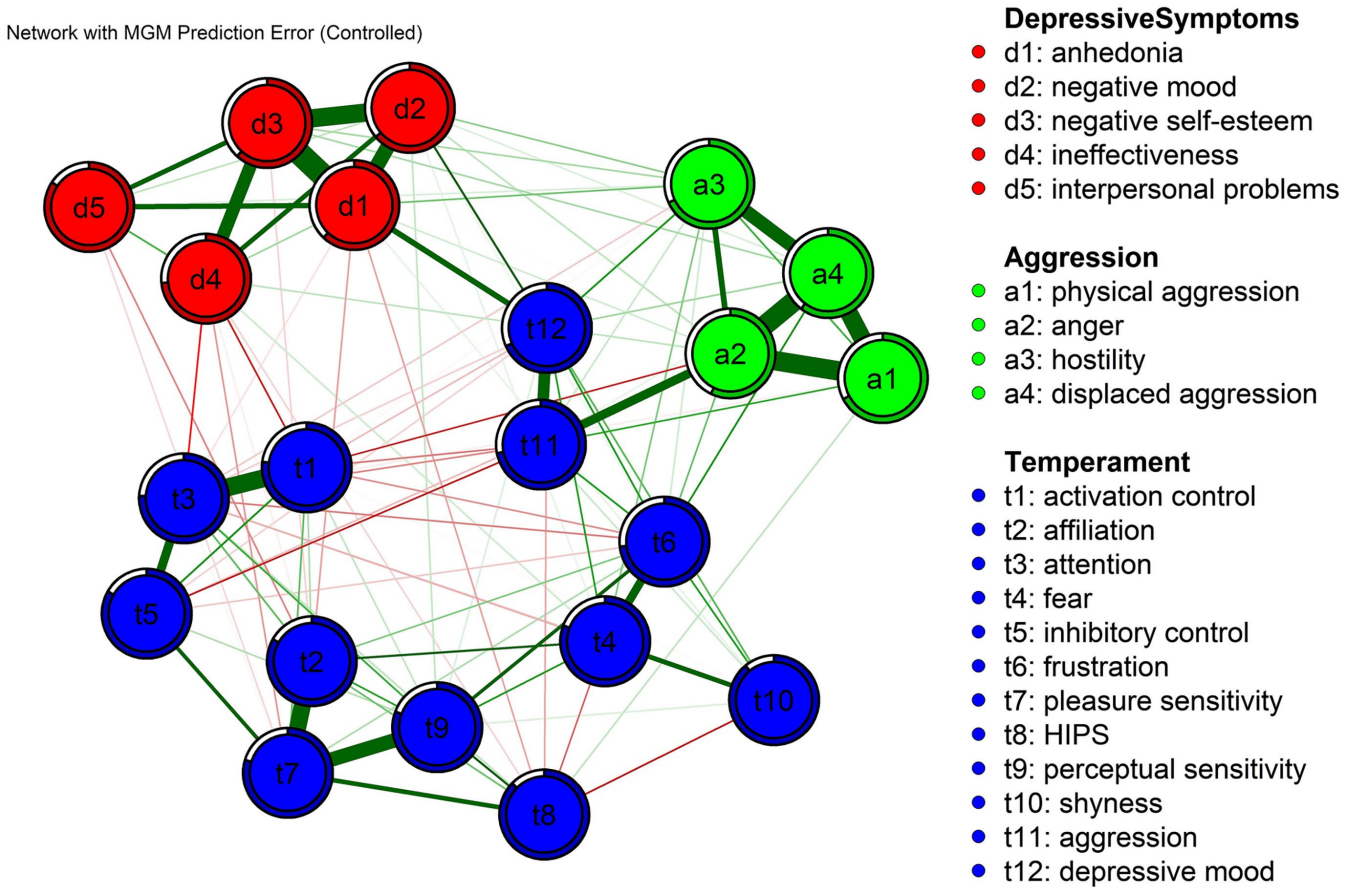
Network structure of preadolescents’ temperament characteristics, internalizing, and externalizing problem behaviors.

Figure 2 illustrates the network structure of preadolescents’ temperament characteristics, alongside internalizing and externalizing problem behaviors. The network comprised 21 nodes and 111 non-zero edges. Green and red edges represent positive and negative partial correlations, respectively, with edge thickness indicating the strength of the association.

Within the temperament community, the strongest positive correlations emerged between activation control and attention; attention and inhibitory control; pleasure sensitivity and affiliation; pleasure sensitivity and perceptual sensitivity; perceptual sensitivity and high-intensity pleasure; and between aggression and depressive mood (Temperament). The strongest negative correlations were found between high-intensity pleasure and shyness, and high-intensity pleasure and fear.

Within the problem behavior domains, extensive positive correlations were observed among the depression sub-dimensions, with notably strong connections between negative self-esteem and anhedonia, and between negative self-esteem and negative mood (Depressive Symptoms). Similarly, strong positive associations were present among the aggression sub-dimensions, particularly between anger and displaced aggression, anger and physical aggression, and displaced aggression and physical aggression.

Between the temperament and depression systems, ineffectiveness was strongly negatively correlated with both activation control and attention, while depressive mood (Temperament) showed strong positive correlations with negative mood (Depressive Symptoms) and anhedonia. Between the temperament and aggression systems, anger was strongly negatively correlated with activation control and strongly positively correlated with aggression. Overall, the network exhibited a distinct community topology, with depression problems clustering with specific temperament dimensions and aggression problems clustering with other temperamental traits.

### 3.3 Node centrality and bridge function

**Figure 3:**
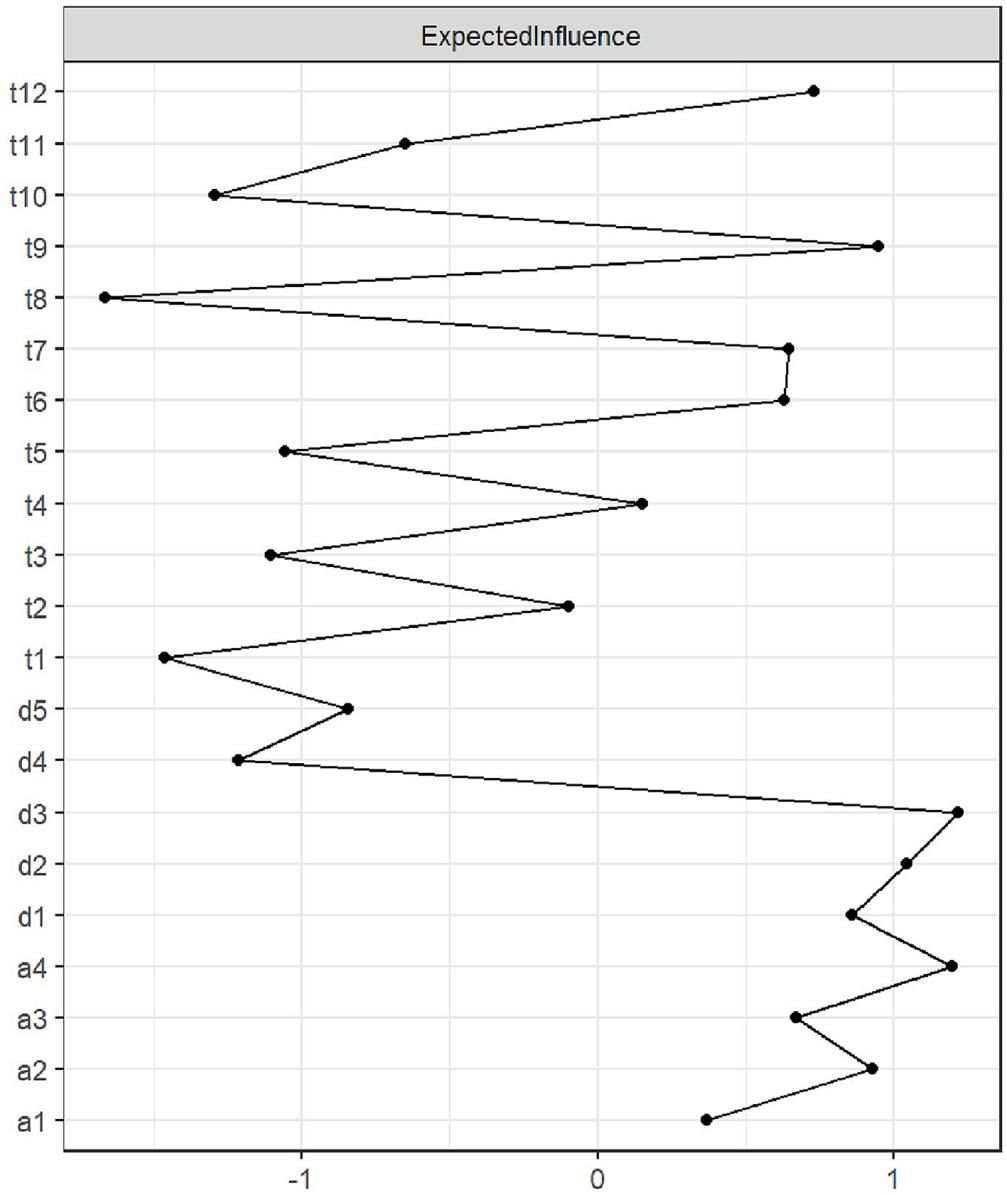
Centrality index: EI.

EI and BEI were calculated for all nodes representing preadolescents’ temperament and problem behaviors. As shown in Figure 3, negative self-esteem (EI = 1.22), displaced aggression (EI = 1.20), and negative mood (Depressive Symptoms; EI = 1.04) exhibited the highest EI values, indicating they hold the highest relative centrality in the network. By contrast, high-intensity pleasure exhibited the lowest EI value (EI = −1.66).

**Figure 4:**
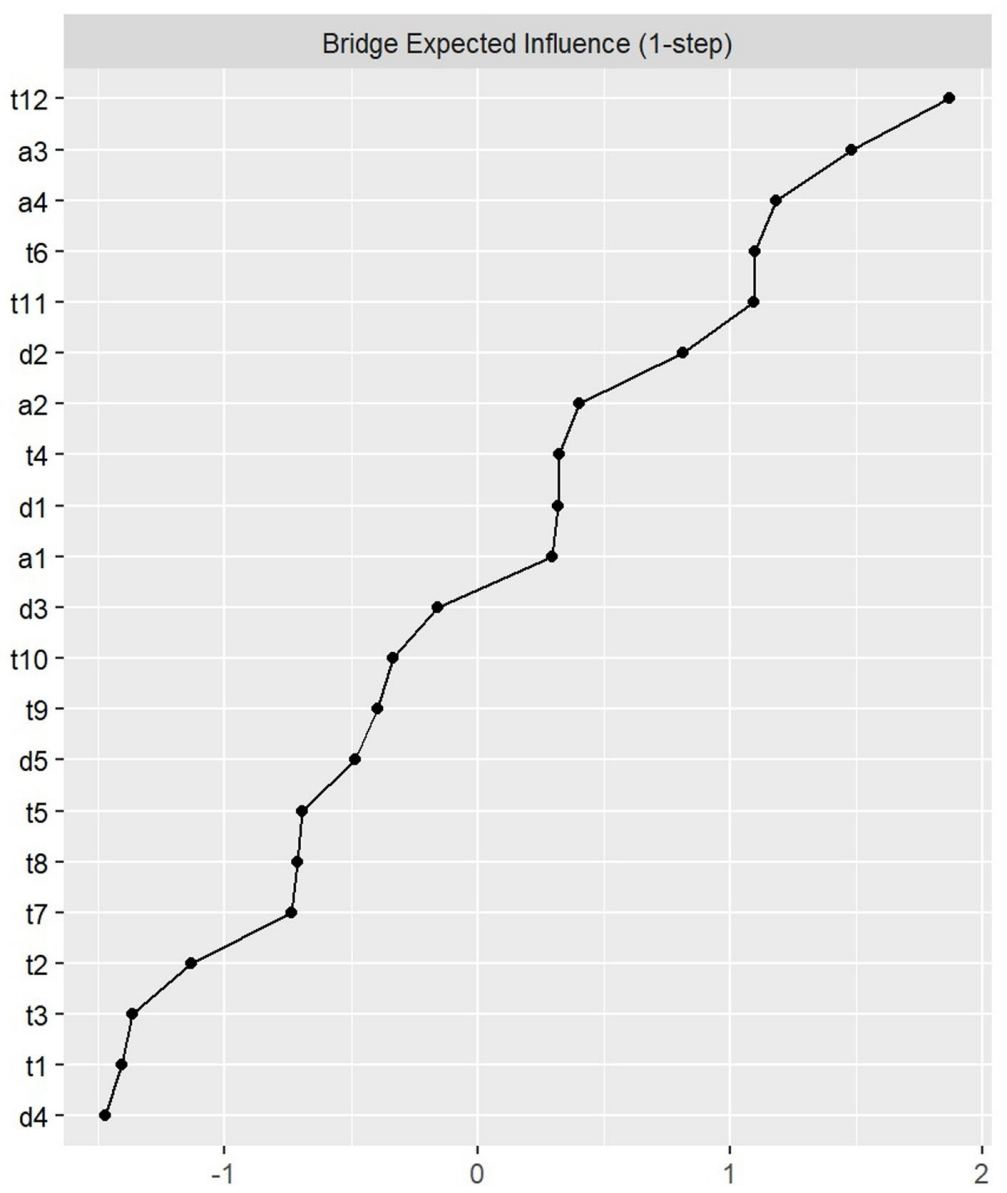
Centrality index: BEI.

As depicted in Figure 4, depressive mood (Temperament; BEI = 1.87) demonstrated the highest BEI, followed by hostility (BEI = 1.48) and displaced aggression (BEI = 1.18). These nodes served as significant bridge hubs connecting the temperament, depression, and aggression communities.

## 4 Discussion

Using a network analysis approach, this study examined the network structure connecting preadolescents’ temperamental characteristics to internalizing (depression) and externalizing (aggression) problem behaviors. EI centrality indices revealed that negative self-esteem was the most central node in the overall temperament-problem behavior network, followed by displaced aggression and negative mood (Depressive Symptoms). This indicates that these nodes were the most strongly connected to the rest of the network, and may thus play a key role in the associations between temperament and problem behaviors.

Negative self-esteem exhibited the highest EI and emerged as the most central node, highlighting the salience of self-evaluative processes during preadolescence. In this transitional period, children become more self-conscious and increasingly sensitive to social comparison and peer evaluation (Grunewald et al., 2022; Willner et al., 2022). Its centrality indicates strong connections with both depressive symptoms and temperamental dimensions, placing it in a highly interconnected position within the overall network. This is consistent with prior evidence that low self-esteem is robustly associated with internalizing distress and externalizing maladjustment in childhood and adolescence (Orth and Robins, 2014; Sowislo and Orth, 2013). Specifically, within the depression community, negative self-esteem was strongly linked to anhedonia and negative mood (Depressive Symptoms), further reflecting its prominent role in this cluster and its extensive connections to other nodes in the broader network.

Displaced aggression exhibited the second highest EI, and its high centrality aligns closely with the developmental characteristics of preadolescence. During this transitional period, social cognitive abilities such as perspective-taking and empathy develop rapidly, making interpersonal frustrations more likely to co-occur with aggressive tendencies (Cheng et al., 2024; Pedersen et al., 2017; Van Heel et al., 2020). However, emotion regulation capacities remain immature, particularly in effortful control mechanisms like attentional shifting and inhibitory control (Eisenberg, 2005). This imbalance between heightened emotional reactivity and insufficient regulatory capacity renders preadolescents susceptible to intense displaced aggression during peer conflicts or perceived unfairness, while struggling to regulate these impulses through effortful control. Meanwhile, heightened sensitivity to external evaluation (Grunewald et al., 2022) and growing self-consciousness may further increase the likelihood of displaced aggression. Notably, displaced aggression also showed elevated bridge expected influence, reflecting robust cross-community connections with temperamental and depressive dimensions.

Furthermore, negative mood (Depressive Symptoms) also exhibited a notably high EI, and its prominent centrality may reflect the developmental characteristic of heightened emotional reactivity during preadolescence. As academic demands and social evaluation intensify (Cheng et al., 2024; Grunewald et al., 2022), state-level negative mood becomes increasingly salient. Within the network, negative mood (Depressive Symptoms) clustered closely with anhedonia and negative self-esteem, while maintaining robust connections to temperamental dimensions. This indicates that negative mood occupies a highly interconnected position between depressive symptoms and temperamental reactivity, reflecting how this affective state manifests in the context of developmental stressors during this transitional period.

Another crucial finding is that depressive mood (Temperament) exhibited the highest bridge expected influence (BEI). Consequently, it emerged as the most central bridge node within the temperament–problem behavior network, connecting the internalizing and externalizing systems. Although depressive mood and negative mood share similar surface labels, they represent distinct constructs. Depressive mood refers to a temperamental disposition characterized by an innate propensity to experience sadness, and it theoretically belongs to Rothbart’s higher-order domain of negative emotionality (Rothbart, 2011). Negative mood, by contrast, reflects a state-like negative affective experience and constitutes a symptom dimension of depression. In essence, depressive mood represents a biologically based temperamental vulnerability, whereas negative mood is its symptomatic expression in specific contexts (Gonda et al., 2020). Because depressive mood (Temperament) represents a biologically based temperamental vulnerability rather than a momentary state, it maintains robust connections across the temperament, depression, and aggression communities. This pattern can be understood through the following theoretical account. High depressive mood (Temperament) reflects a temperamental disposition that, in the context of developmental stressors, is associated with heightened depressive symptoms (Zheng et al., 2024). This affective state may be expressed bidirectionally: internalizing as depressive symptoms (anhedonia, worthlessness) or externalizing as hostility and anger—both pathways have been found to be associated with deficits in effortful control (Rothbart, 2011). Notably, according to Conway et al. (2024), state-level negative mood can be understood as the contextual, momentary expression of this depressive mood (Temperament). Collectively, these findings align with Rothbart’s model, highlighting how interactions among negative emotionality, effortful control, and environmental stressors may together be associated with problem behavior trajectories.

Hostility also exhibited a notably high bridge expected influence, emerging as another crucial bridge node connecting the temperament, depression, and aggression communities. From the perspective of preadolescent social-cognitive development, the rapid expansion of social circles and the growing salience of peer relationships during this stage may heighten preadolescents’ sensitivity to interpersonal cues (Grunewald et al., 2022; Willner et al., 2022). However, their capacity for self-reflection and cognitive error correction remains highly unstable (Willner et al., 2022). Furthermore, depressive mood (Temperament) and predispositions toward frustration may contribute to a tendency for preadolescents to make hostile attributions by default when decoding ambiguous social cues (Crick and Dodge, 1994). Compounded by heightened self-consciousness and limited social experience, preadolescents may find it challenging to revise these erroneous inferences.

The network structure further elucidated several other crucial associations between temperament and problem behaviors. Notably, high-intensity pleasure exhibited the lowest, negative EI value, indicating an inhibitory effect on other nodes. This suggests that higher high-intensity pleasure is associated with lower levels of shyness and fear, reflecting the inherent tension between approach motivation (i.e., sensation-seeking) and behavioral inhibition (i.e., social risk avoidance) (Roxburgh et al., 2022). In early adolescence, youth exhibit a heightened drive for exploration while simultaneously becoming increasingly sensitive to peer evaluations. This developmental paradox—in which the motivation to approach novelty coexists with heightened concerns about social evaluation—may be related to the strength of the negative association observed between these opposing traits (Hassan et al., 2021).

Moreover, strong negative correlations emerged between dimensions of effortful control (i.e., activation control and attention) and the ineffectiveness dimension of depression, as well as between anger and activation control. Ineffectiveness reflects a lack of confidence in one’s abilities and a perceived powerlessness to achieve goals, serving as a manifestation of negative self-evaluation within depressive symptoms. During preadolescence, when academic performance emerges as a primary domain of daily functioning, preadolescents with deficits in executive functioning are more susceptible to academic setbacks (Roebers et al., 2012). When combined with negative cognitive schemas, these experiences of failure may be related to a pervasive sense of ineffectiveness (Vierikko et al., 2026). The negative correlation between anger and activation control aligns with previous findings that poor impulse control is associated with aggressive behavior. This is consistent with previous evidence indicating that low effortful control and high anger jointly predict externalizing problems (Eisenberg et al., 2007).

Several limitations of the current study warrant consideration. First, the cross-sectional nature of the data precludes causal inferences. Future research should use longitudinal network modeling to capture the dynamic temporal interplay between temperamental traits and problem behaviors. Second, To enhance generalizability, future studies should recruit broader and more diverse samples—including various age cohorts and sociocultural backgrounds—to facilitate cross-cultural comparisons. Third, the exclusive reliance on self-report measures introduces the risk of social desirability and response biases. Subsequent research should adopt a multi-informant, multi-method approach that integrates parent ratings, behavioral tasks, and physiological indices (e.g., EEG-derived functional connectivity) to yield more objective and comprehensive assessments. Finally, the present study did not account for potential age-related variations. Given the rapid maturation of cognitive reappraisal skills during preadolescence, future investigations should explore how the topological structure of these problem behavior networks shifts across different developmental stages.

The present findings suggest that these identified hub nodes may represent important underlying mechanisms linking temperament and problem behaviors. Whether interventions targeting these nodes can lead to broader preventive effects across the symptom network remains to be tested in future longitudinal and intervention research.

## 5 Conclusion

This study employed network analysis to examine the associations between temperament and problem behaviors. The results revealed the following descriptive features: (1)negative self-esteem exhibited the highest expected influence, followed by displaced aggression and negative mood (Depressive Symptoms); (2)depressive mood (Temperament) exhibited the highest bridge expected influence, emerging as the primary bridge node connecting the internalizing and externalizing systems; and (3)hostility and displaced aggression showed elevated bridge expected influence, serving as significant bridge hubs connecting the temperament, depression, and aggression communities. Our findings provide an initial characterization of the conditional association patterns among these variables.

## Data Availability

The raw data supporting the conclusions of this article will be made available by the authors, without undue reservation.
